# Pollution Problem in River Kabul: Accumulation Estimates of Heavy Metals in Native Fish Species

**DOI:** 10.1155/2015/537368

**Published:** 2015-08-03

**Authors:** Habib Ahmad, Ali Muhammad Yousafzai, Muhammad Siraj, Rashid Ahmad, Israr Ahmad, Muhammad Shahid Nadeem, Waqar Ahmad, Nazia Akbar, Khushi Muhammad

**Affiliations:** ^1^Department of Genetics, Hazara University, Mansehra 21300, Pakistan; ^2^Department of Zoology, Islamia College, Peshawar 25000, Pakistan; ^3^Department of Zoology, Hazara University, Mansehra 21300, Pakistan; ^4^Department of Chemistry, Malakand University, Chakdara 18800, Pakistan

## Abstract

The contamination of aquatic systems with heavy metals is affecting the fish population and hence results in a decline of productivity rate. River Kabul is a transcountry river originating at Paghman province in Afghanistan and inters in Khyber Pakhtunkhwa province of Pakistan and it is the major source of irrigation and more than 54 fish species have been reported in the river. Present study aimed at the estimation of heavy metals load in the fish living in River Kabul. Heavy metals including chromium, nickel, copper, zinc, cadmium, and lead were determined through atomic absorption spectrophotometer after tissue digestion by adopting standard procedures. Concentrations of these metals were recorded in muscles and liver of five native fish species, namely, *Wallago attu*, *Aorichthys seenghala*, *Cyprinus carpio*, *Labeo dyocheilus*, and *Ompok bimaculatus*. The concentrations of chromium, nickel, copper, zinc, and lead were higher in both of the tissues, whereas the concentration of cadmium was comparatively low. However, the concentration of metals was exceeding the RDA (Recommended Dietary Allowance of USA) limits. Hence, continuous fish consumption may create health problems for the consumers. The results of the present study are alarming and suggest implementing environmental laws and initiation of a biomonitoring program of the river.

## 1. Introduction

River Kabul is the largest river of Hindu Kush Mountains. It serves as the main drainage basin of most of the valleys of Hindu Kush Mountains. The River is fed by two main tributaries, that is, River Chitral and River Swat. It originates from the base of the Unai Pass [1] in the Paghman Mountains in Afghanistan, whereas the rivers Chitral and Swat originate from Mastuj Mountains and Hindu Raj Mountains of Chitral and Swat mountains in Pakistan, respectively. The River provides habitat for the variety of life forms, including 54 fish species, mainly belonging to the Carp and Mystus families; out of which some of the species, for example,* Botia rostrata*, are endemic to River Kabul Pakistan (reviewed in [2, 3]).

The risk issues, assessment, and maintaining the quality of environment are the main focus of the researchers. The estimation and monitoring of environmental pollution are becoming increasingly important to develop different monitoring methods and strategies (reviewed in [4]). Heavy metal pollution is a major environmental concern issue, which is mainly contributed by sewage and industrial waste and agricultural run-off. Natural phenomena such as earthquake, landslides, tornadoes, cyclones, and weathering of rocks also contribute towards heavy metal pollution. Heavy metals such as lead, cadmium, copper, and nickel present in the aquatic environment have accumulated in fish body which have led to toxic events in the past [5]. More than 72 small- and large-scale industries are discharging their untreated effluents to River Kabul [6], which certainly is affecting the quality of water and thereby the fish health and production. Less information is available about heavy metals load in edible fishes of River Kabul.

Our study aimed to assess and determine the chromium, nickel, copper, zinc, cadmium, and lead concentrations in the muscles and liver of five common edible fish species like Mulee* (Wallago attu*), Singhara (*Aorichthys seenghala)*, common carp (*Cyprinus carpio*), torki (*Labeo dyocheilus*), and Shermai (*Ompok bimaculatus*).

## 2. Materials and Methods

### 2.1. Sampling Procedure

Five fish species, namely,* Wallago attu*,* Ompok bimaculatus*,* Labeo dyocheilus*,* Cyprinus carpio*, and* Aorichthys seenghala*,* of different sizes and weights* were caught from River Kabul during late night with the help of local fisher men through Gill Net of 40 × 6 ft. with a cork line at the top rope and metal line with the ground rope made locally of nylon wire. Sampling was carried out downstream Nowshera city where river becomes narrow and the pollutants concentration rises after city effluent discharges. Fish samples were temporarily stored in iceboxes and identified by the procedures described by Butt and Mirza [7]. Fishes were washed with distilled water and dissected for sampling of various tissues. Weighed portions (2.0 g) of the tissue (muscle and liver) were excised and kept in labeled sterilized polythene sampling bags in the deep freezer at −20°C.

### 2.2. Estimation of Heavy Metals

For estimation of heavy metals, the tissue samples were thawed, rinsed in distilled water, and blotted and known weights (0.2 g) were shifted to 100 mL sterilized volumetric flasks. The samples were digested according to the modified protocol of van den Heever and Frey [8] and Yousafzai and Shakoori [9]. The modification was that, instead of putting 10 mL nitric acid (55%) and 5.0 mL perchloric acid (70%) at the time of digestion, 5.0 mL nitric acid (55%) and 1.0 mL perchloric acid (70%) were added to each sample and the flasks were kept lid tight overnight. After 24 h, 5.0 mL nitric acid (55%) and 4.0 mL perchloric acid (70%) were added to each flask. The flasks were then placed on a hot plate and allowed to digest at 200 to 250°C until a transparent solution was obtained. After digestion, samples were cooled and diluted to 10.0 mL with distilled water. Determination of heavy metals, that is, nickel, copper, zinc, cadmium, and lead, was done by atomic absorption spectrophotometer (Spectra-AA-700). Three different fishes of the same species were analyzed and the results were the average of triplicate independent measurements. Mean and standard error of mean values of estimates were recorded and compared. The standard conditions were set on atomic absorption spectrophotometer during analysis (Table 1).

A range of analytical standards for each metal was prepared from E. Merck stock solution. Standard curves were prepared and the ODs (optical densities) obtained were calibrated against the standard curves to know the concentration of heavy metals.

## 3. Results and Discussions

Results show that accumulation of heavy metals adversely affects liver and muscles, which directly affect the growth and development of fishes. Muscle is suggested to be the major tissue of interest for monitoring of environmental contamination with metals [9]. Liver in fish plays a protective role against metal exposure, by acting as a storage site and being a vital organ in the regulation of metals. Large amount of metallothioneins (MTs) protein induction occurs in the liver tissue of fish thus acting as a major sequestering organ. Therefore, the liver is considered as one of the major metal bioaccumulation organs [10]. Histopathological lesions of liver proved to be the most sensitive and reliable indicators of metal exposure [11].

Results obtained for accumulation of heavy metals in muscle and liver tissues of the fish species are summarized in Tables 2 and 3, respectively. Our findings revealed that the accumulation profile was highly variable among different species and different samples of the same species. Variations in accumulation of metals were even recorded in different tissues of the 96 same fish. These findings are in conformity of similar studies carried out elsewhere [12, 13]. The presence of high metal concentrations in fishes can simply be correlated to the presence of higher metal concentration in water and sediments of River Kabul as reported by Yousafzai and Shakoori [9] and Trevor and Stephan [6]. According to Trevor and Stephan [6], the concentration of chromium in ambient water downstream Nowshera ranged 0.068–0.64 mg/L, Zn ranged 0.35–0.52 mg/L, Co ranged 0.38–0.48 mg/L, Ni ranged 0.04–0.074 mg/L, and Pb ranged 0.006–0.141 mg/L. The comparison of our findings with that of Yousafzai and Shakoori [9] showed the levels of heavy metal accumulation enhanced with the passage of time. This reflects that in the past few years a further increase in the level of heavy metals has occurred in the river water, which is suggestive of the implementation of proper environmental laws and a biomonitoring program for the river.

Natural water may receive chromium from the industrial effluents [14]; once absorbed, it passes into the blood and is distributed to various organs, particularly the liver. The liver serves as a primary storage and detoxification site for chromium. In the liver, chromium is stored, linked to proteins smaller peptides, and induces changes in blood and tissue metabolism with acute and chronic poisoning leading to hyperglycemia and glycogenolysis in the brain and liver.

Mean values of the accumulated Cr in muscles of* W. attu*,* A. seenghala*,* C. carpio*,* L. dyocheilus*, and* O. bimaculatus* were 533.3 ± 206.1, 565.3 ± 148.7, 489.0 ± 49.7, 647.3 ± 105.1, and 703.0 ± 125.3 while its concentration in liver was 513.0 ± 159.7, 619.0 ± 161.9, 493.7 ± 56.5, 643.7 ± 64.9, and 670.7 ± 182.0 *μ*g/g wet weight, respectively (Tables 2 and 3). The Cr concentration in tissues of fish species was in the order of* O. bimaculatus* >* L. dyocheilus* >* A. seenghala* >* W. attu* >* C. carpio*. This shows that the metal accumulation is highest in* O. bimaculatus* and lowest in* C. carpio*. Bhattacharya et al. [15] have reported highest level of Cr in muscle tissue of commercially edible fishes from upper stretch of the Ganga River at West Bengal, India. Similarly, Yilmaz [16] has reported 1.46 and 1.28 *μ*g/g of Cr in the muscle of* Mugil cephalus* and* Trachurus mediterraneus* respectively in some fishes caught from Turkey. In another study Olaifa et al. [17] reported 0.07 ppm of Cr in the muscle of* Clarias gariepinus* got from Eleiyele Lake and Zartech fish farm in Ibandan, Nigeria. Türkmen et al. [18] have also recorded 0.07–6.46 mg/g k^−1^ of Cr in three commercially valuable fish species,* Saurida undosquamis*,* Spamrus aurata* and* Mullus baratus* from Iskenderun Bay, North East Mediterranean Sea, Turkey. Demirezen and Uruç [19] have reported 8.44 and 9.51 (*μ*g/g) of Cr in certain fishes from Kayseri, Turkey. Yousafzai and Shakoori [9] recorded high levels of Cr 5.90 ± 0.04 and 3.2 ± 0.05 *μ*g/g wet weight, in muscle and liver of* Tor putitora* from River Kabul. Our findings suggest that River Kabul has more concentration of Cr as compared to other water bodies, mentioned above, and the concentration of Cr is comparatively high. The accumulation of heavy metals in the muscle of coastal fishes has been studied and predominant bioaccumulation of Fe, Zn, and Cu was recorded by Kumar et al. [20].

Nickel is capable of producing severe physiological changes during the past few years, causing failure of the respiratory mechanism resulting in enhanced rates of mortality of the fish species [21]. Chronic low level of nickel exposure can cause serious lung damage, birth defects, kidney diseases, lung cancer, and so forth [22]. Mean values of Ni in muscles of* W. attu*,* A. seenghala*,* C. carpio*,* L. dyocheilus*, and* O. bimaculatus* were 106.7 ± 6.8, 94.7 ± 33.3, 74.7 ± 17.3, 117.7 ± 33.5, and 135.0 ± 52.6 (Table 2). In livers, however, its concentration was 108.0 ± 19.9, 117.7 ± 13.3, 80.0 ± 16.1, 111.7 ± 21.4, and 100.3 ± 66.8 *μ*g/g wet weight, respectively (Table 2). Ni concentration in muscles of different fish species was in the order of* O. bimaculatus* >* L. dyocheilus* >* W. attu* >* A. seenghala* >* C. carpio.* This shows that the bioaccumulation of Ni was highest in the muscle of* O. bimaculatus* and lowest in* C. carpio*. In the liver, it was in the order of >* seenghala* >* dyocheilus* >* attu* >* bimaculatus* >* carpio*. This shows that the Ni absorption is lowest in the liver of* carpio* and highest in* seenghala.* The wide difference in bioaccumulation might be due to herbivorous nature of* carpio*. Being herbivorous, it is less exposed to metal bioaccumulation. Yilmaz [16] has reported 1.22 and 0.94 *μ*g/g of Ni in the wet weight muscles of* Mugil cephalus* and* mediterraneus*, respectively, caught in Iskenderun Bay, Turkey. Yousafzai and Shakoori [9] have also reported high level of Ni 87 ± 6.04, 113 ± 11.74 *μ*g/g wet weight, in muscle and liver of the fish,* putitora,* caught from the same water body. Bioaccumulation of Ni in all the fishes surpasses the RDA limits of 10 *μ*g/100 g (Table 4).

Copper is an essential element, but it is toxic in excess. From a dietary perspective, the primary toxic action is predominantly the production of free radicals in tissues where copper accumulates. In addition, dietary copper toxicity can occur at several other loci in the gut and includes inhibition of digestive enzymes and reduced gut motility [23].

Variation of copper accumulation in the muscle and liver of fish species is presented in Tables 2 and 3. In muscles, the bioaccumulation varied in the order of* C. carpio* >* O. bimaculatus* >* L. dyocheilus* >* A. seenghala* >* W. attu*, which shows that its accumulation was highest in the muscle of* C. carpio* and lowest in* W. attu*. Bhattacharya et al. [15] have reported 1.45 and 1.29 *μ*g/g wet weights of Cu in the muscles of the fish species,* cephalus* and* mediterraneus*, respectively. Yap et al. [24] have also recorded 1.98 *μ*g/g wet weight of Cu in the edible flesh (muscles)* Oreochromis mossambicus*. Different studies [9, 25–27] concluded that the concentration of Cu varies significantly in different species of fish species. Cu in liver of* W. attu* was generally with a mean value of 136.0 ± 9.6, in* A. seenghala* was 220.7 ± 8.2, in* A. carpio* was 390.0 ± 13.5,* L. dyocheilus* was 1644.0 ± 691.6, and* Ompok bimaculatus* was with mean value of 164.0 ± 101.3 *μ*g/g of the wet weight. Cu concentration in liver was however in the order of* dyocheilus* >* carpio* >* seenghala* >* bimaculatus* >* attu.* This shows that the metal bioaccumulation was highest in the liver of* dyocheilus* and lowest in* bimaculatus*. Similar results were observed by Subathra and Karuppasamy [26] who recorded 82.12 and 70.65 *μ*g/g of Cu in the liver of different sizes fingerlings and adult of healthy* Mystus vittatus.* However, Ruelas-Inzunza and Páez-Osuna [27] reported 48.6 *μ*g/g of Cu in liver of sperm whale (*Physeter catodon*). In another study, Clearwater et al. [28] have reported 667 ± 81 (*μ*g kg^−1^) of Cu in liver of rainbow trout. Although the situation of Copper in the fish species of River Kabul is alarming, it is not the worst like other heavy metals.

Zinc generally enters the fish bodies via gills, general body surface, and alimentary canal and its excretion takes place mainly via the gastrointestinal tract. Mean values for the concentration of Zn in the muscles of* W. attu*,* A. seenghala*,* C. carpio*,* L. dyocheilus*, and* O. bimaculatus* were 649.0 ± 107.0, 1167.7 ± 230.8, 826.3 ± 166.6, 883.0 ± 185.3, and 902.0 ± 112.8 *μ*g/g, respectively, in the wet weight of the muscles (Table 1). The concentration of Zn was in the order of* A. seenghala* >* O. bimaculatus* >* L. dyocheilus* >* C. carpio* >* W. attu*. This shows that the metal bioaccumulation was highest in the muscle of* A. seenghala* and lowest in* W. attu*. Yilmaz [16] recorded 38.23 and 19.55 *μ*g/g wet weight of Zn in the muscle of fish species, namely,* Mugil cephalus* and* Trachurus mediterraneus*, respectively, caught from Iskenderun Bay, Turkey. Similarly Yap et al. [24] got 58.4 *μ*g/g wet weight of Zn in the muscles of Tilapia fish (*Oreochromis mossambicus*) caught from a pond in Kelana Jaya. In a report, Ruelas-Inzunza and Páez-Osuna [27] recorded 388 *μ*g/g of Zn in muscle of gray whale (*Eschrichtius robustus*), which is reasonably less than the values we recorded for fishes of River Kabul.

In the present study,* Wallago attu* accumulated Zn with mean value of 509.7 ± 95.8,* seenghala* was 2279.7 ± 1614.9,* carpio* was 3319.0 ± 376.8, and* dyocheilus* was 1175.7 ± 649.0 and* bimaculatus* had the mean value of 860.0 ± 160.8 *μ*g/g wet weight. Zn concentration in liver was, however, in the order of* carpio* >* seenghala* >* dyocheilus* >* bimaculatus* >* attu*. This shows that the metal bioaccumulation was the highest in the liver of* Cyprinus carpio* and lowest in* Wallago attu.* Previously, Subathra and Karuppasamy [26] have reported 388 *μ*g^−1^ of Zn in liver of* Eschrichtius robustus.* The highest Zn concentration was found in the liver of* Clarias gariepinus* in treated sewage water [8]. Annune and Lyaniwura [29] reported that the liver of* niloticus* and* gariepinus* accumulated Zn more than other tissues. Similarly, van den Heever and Frey [8] had reported Zn 1060.57 ± 14.61 and 1935.5 ± 70.89 *μ*g/g wet weight of Zn in muscle and liver of fish* putitora* caught from the Kabul River. The results reveal that River Kabul is more polluted with Zn as compared to all the other water bodies, mentioned here.

Cadmium an anthropogenic metal pollutant is extremely toxic to aquatic animals with a long biological half-life and can produce both hepatic and renal injuries in mammals and fish [30]. Its access causes coronary artery disease, hypertension, emphysema, and chronic pulmonary diseases. Cadmium remains and accumulates in the respective uptake tissue during waterborne or dietary exposure but has also been shown to enter the circulation and accumulate to a significant extent in the liver [31]. The accumulation of Cd in the muscles and livers is given in Tables 1 and 2, respectively. Its mean values in muscles of* W. attu*,* A. seenghala*,* C. carpio*,* L. dyocheilus*, and* O. bimaculatus* were 68.0, 60.7, 53.3, 66.7, and 71.7 *μ*g/g, respectively. However, in muscles its bioaccumulation was in the order of* O. bimaculatus* >* W. attu* >* L. dyocheilus* >* A. seenghala* >* C. carpio*. Its accumulation was highest in the muscle of* O. bimaculatus* and lowest in* C. carpio*. In the livers, mean values of Cd were 64.3, 64.7, 58, 72.3, and 138.3 *μ*g/g wet weight, in the order of* bimaculatus* >* dyocheilus* >* seenghala* >* Wallago attu* >* Cyprinus carpio*. This shows that the metal bioaccumulation was highest in the liver of* bimaculatus* and lowest in* carpio*. In previous studies, Türkmen et al. [18] obtained 0.01–4.16 (*μ*g/kg^−1^) of Cd in three commercially valuable fish species, namely,* Saurida undosquamis*,* S. aurata*, and* Mullus barbatus* of Turkey. In another study, Yap et al. [24] recorded 2.42 *μ*g/g of Cd in muscles of* Oreochromis mossambicu* from a Kelana Jaya Pond. Made changes accordingly. Kumar et al. [20] have also reported 0.77 and 1.04 *μ*g/g of Cd in certain fishes of Kayseri, Turkey. Bhattacharya et al. [15] recorded that muscle tissue accumulated more cadmium than gonads and skin of fishes. Our report shows a higher concentration of Cd in fishes of the Kabul River, as compared to the recommended international standards (Table 2). This study reports that River Kabul is more polluted with Cd as compared to that from different parts of the world.

Lead enters the aquatic environment through erosion and leaching from the lead dust fallout, combustion of gasoline, and municipal and industrial discharges. Lead is a leading cause of birth defects, cardiovascular disease, hypertension, neurological disease, kidney disease, learning disability, retardation, tooth cavities, and so forth [32]. Devi and Banerjee [33] reported reduction in serum protein contents of* Aristichthys nobilis* following exposure to lead. Lead Concentration in the muscle of* attu* was 599.3 ± 188.3,* seenghala* was 350.7 ± 37.2,* carpio* was 226.3 ± 222.2,* dyocheilus* was 528.7 ± 236.4, and* bimaculatus* was 407.0 ± 126.6 *μ*g/g wet weight. The concentration of Pb in muscle of different fish species was in the order of* attu* >* dyocheilus* >* bimaculatus* >* seenghala* >* carpio*, reflecting highest bioaccumulation in* attu* and lowest in* carpio*. While in livers* attu* had 623.3 ± 276.5,* seenghala* had 240.0 ± 104.2,* carpio* had 261.3 ± 72.7,* dyocheilus* had 377.0 ± 300.2, and* bimaculatus* had 1390.0 ± 1530.2 *μ*g/g wet weight of lead. Its concentration however varied in the order of* bimaculatus* >* attu* >* dyocheilus* >* carpio* >* seenghala*. This shows that the metal bioaccumulation is the highest in the liver of* bimaculatus* and lowest in* seenghala*. In earlier studies Ruelas-Inzunza and Páez-Osuna [27] have reported 0.9 and 4.2 *μ*g/g of Pb in muscle of the gray whale and liver of sperm whale whereas Yilmaz [16] has recorded 7.45 and 1.3 *μ*g/g wet weights of Pb in the muscle of* Mugil cephalus* and* Trachurus mediterraneus*, respectively. Yousafzai and Shakoori [9] had also reported 227.4 ± 20.44 and 136.8 ± 9.08 *μ*g/g wet weight of Pb in muscle and liver of* Tor putitora* caught from River Kabul.

## 4. Conclusion

Different countries have their standards of maximum permissible limits of different metals in the fish muscle with some variations depending upon the environmental conditions. Unfortunately in Pakistan such standards are not yet established or if prepared are not available to researchers. Therefore, the results relating to different metals in the fish muscle are compared with the U.S. Recommended Dietary Allowance (RDA) supplied by hundred grams (100 g) serving fish muscle to prove the contention. Out of six heavy metals, Zn, Ni, Cr, Cd, and Pb are exceeding the RDA limits, while copper remains within the limits.

This shows that continuous fish consumption is not suitable for human health. However, as the fish is very rarely consumed by the people here due to many reasons, high health risk has not yet been identified and reported.

## Figures and Tables

**Table 1:** The standards used to analyze the variable using atomic absorption spectrophotometer (Spectra-AA-700).

Element	Wavelength (nm)	Flame gases (A-AC air acetylene)	Lamp current (m-ampere)	Band pass
Cr	357.9	A-AC	10	0.5
Cu	324.7	A-AC	5	0.5
Pb	283.3	A-AC	10	0.5
Ni	232.0	A-AC	15	0.2
Zn	213.9	A-AC	8	0.5

**Table 2:** Metal concentration (*μ*g/g wet weight) in liver and muscles of selected edible fish species of River Kabul.

Serial number	Fish species	Metal	Liver (*n* = 3) mean ± S.E	Muscle (*n* = 3) mean ± S.E
1	*Wallago attu *	Chromium	509.7 ± 95.8	533.3 ± 206.1
		Nickel	108.0 ± 19.9	106.7 ± 6.8
		Copper	513.0 ± 159.7	46.3 ± 29.0
		Zinc	136.0 ± 9.6	649.0 ± 107.0
		Cadmium	64.3 ± 7.6	68.0 ± 15.0
		Lead	623.3 ± 276.5	599.3 ± 188.3

2	*Aorichthys seenghala *	Chromium	2279.7 ± 1614.9	565.3 ± 148.7
		Nickel	117.7 ± 13.3	94.7 ± 33.3
		Copper	619.0 ± 161.9	132.7 ± 13.4
		Zinc	220.7 ± 8.2	1167.7 ± 230.8
		Cadmium	64.7 ± 14.0	60.7 ± 17.2
		Lead	240.0 ± 104.2	350.7 ± 37.2

3	*Cyprinus carpio *	Chromium	3319.0 ± 376.8	489.0 ± 49.7
		Nickel	80.0 ± 16.1	74.7 ± 17.3
		Copper	493.7 ± 56.5	303.0 ± 255.8
		Zinc	390.0 ± 13.5	826.3 ± 166.6
		Cadmium	58.0 ± 2.9	53.3 ± 2.9
		Lead	261.3 ± 72.7	226.3 ± 222.2

4	*Labeo dyocheilus *	Chromium	1175.7 ± 649.0	647.3 ± 105.1
		Nickel	111.7 ± 21.4	117.7 ± 33.5
		Copper	643.7 ± 64.9	191.7 ± 30.6
		Zinc	1644.0 ± 691.6	883.0 ± 185.3
		Cadmium	72.3 ± 10.3	66.7 ± 8.5
		Lead	377.0 ± 300.2	528.7 ± 236.4

5	*Ompok bimaculatus *	Chromium	860.0 ± 160.8	703.0 ± 125.3
		Nickel	100.3 ± 66.8	135.0 ± 52.6
		Copper	670.7 ± 182.0	241.3 ± 40.1
		Zinc	164.0 ± 101.3	902.0 ± 112.8
		Cadmium	138.3 ± 93.7	71.7 ± 12.1
		Lead	1390.0 ± 1530.2	407.0 ± 126.6

**Table 3:** Heavy metals concentrations *μ*g/g in liver samples of edible fish species of River Kabul.

Fish	Metals	Sample number			Mean	Standard deviation
		1	2	3		
*Wallago attu *	Zinc	385	618	526	509.7	509.7 ± 95.8
	Nickel	80	125	119	108.0	108.0 ± 19.9
	Chromium	731	559	249	513.0	513.0 ± 159.7
	Copper	133	126	149	136.0	136.0 ± 9.6
	Cadmium	72	67	54	64.3	64.3 ± 7.6
	Lead	723	901	246	623.3	623.3 ± 276.5

*Aorichthys seenghala *	Zinc	4553	1333	953	2279.7	2279.7 ± 1614.9
	Nickel	112	136	105	117.7	117.7 ± 13.3
	Chromium	598	827	432	619.0	619.0 ± 161.9
	Copper	231	211	220	220.7	220.7 ± 8.2
	Cadmium	59	84	51	64.7	64.7 ± 14.0
	Lead	120	374	226	240.0	240.0 ± 104.2

*Cyprinus carpio *	Zinc	3487	2797	3673	3319.0	3319.0 ± 376.8
	Nickel	102	64	74	80.0	80.0 ± 16.1
	Chromium	462	573	446	493.7	493.7 ± 56.5
	Copper	371	398	401	390.0	390.0 ± 13.5
	Cadmium	57	62	55	58.0	58.0 ± 2.9
	Lead	174	352	258	261.3	261.3 ± 72.7

*Labeo dyocheilus *	Zinc	842	602	2083	1175.7	1175.7 ± 649.0
	Nickel	121	132	82	111.7	111.7 ± 21.4
	Chromium	658	715	558	643.7	643.7 ± 64.9
	Copper	1143	1167	2622	1644.0	1644.0 ± 691.6
	Cadmium	74	84	59	72.3	72.3 ± 10.3
	Lead	797	221	113	377.0	377.0 ± 300.2

*Ompok bimaculatus *	Zinc	710	1083	787	860.0	860.0 ± 160.8
	Nickel	43	194	64	100.3	100.3 ± 66.8
	Chromium	545	928	539	670.7	670.7 ± 182.0
	Copper	42	290	160	164.0	164.0 ± 101.3
	Cadmium	269	92	54	138.3	138.3 ± 93.7
	Lead	3545	141	484	1390.0	1390.0 ± 1530.2

**Table 4:** US Recommended Daily Dietary Allowance (RDA) supplied by a 100 g of fish muscle.

Metals	Concentration (mg)	Microgram (*μ*g)
Zinc	2.6	2600
Nickel	0.01	10
Chromium	0.05–0.20	50–200
Copper	2.0–3.0	2000–3000
Cadmium	0.014	14
Lead	0.3	300
